# Penetration and durability of CPP-ACP paste and sodium fluoride varnish as desensitizing agents: An in vitro comparison

**DOI:** 10.34172/joddd.2023.28050

**Published:** 2023-07-17

**Authors:** Naghmeh Golriz, Mehrdad Barekatain, Parvin Mirzakocheki Broujeni

**Affiliations:** Department of Operative Dentistry, Isfahan (Khorasgan) Branch, Islamic Azad University, Isfahan, Iran

**Keywords:** Dentin, Sensitivity, Durability, Fluoride varnish, Scanning electron microscopy

## Abstract

**Background.:**

This study aimed to investigate and compare the penetration and durability of two dentin desensitizers, sodium fluoride varnish and casein phosphopeptide-amorphous calcium phosphate (CPP-ACP) paste, using electron microscopy.

**Methods.:**

The study was performed on 60 dentin specimens prepared from extracted human premolars. After applying 17% EDTA to remove the smear layer, the specimens were divided into two groups. MI Paste and Bi-fluoride varnish were applied to the specimens. Microscopic images of 20 samples were obtained immediately. Twenty other samples were accessed after 15 days, and the other 20 were accessed after 30 days of toothbrushing and thermal cycling. Both surface and longitudinal cross-sectional images (after sample fracture) were studied. Data were analyzed with two-way ANOVA and Mann-Whitney U test at a significance level of *P*<0.05.

**Results.:**

The mean depth of material penetration was significantly time-dependent and fluctuated in both groups. There was a significant difference between the mean level of plugs between 0, 15, and 30 days (*P*<0.001). Penetration increased with time for the MI Paste group, while in the Bi-fluoride group, the increase was significant at 15 and 30 days than immediately after application. The mean thickness of the plugs was significantly different at the three time periods, and MI Paste showed a sudden decrease in plug thickness after 15 days.

**Conclusion.:**

Immediately after application, Bi-fluoride occluded dentinal tubules more effectively, and its durability after abrasion and thermal fatigue were higher than MI Paste.

## Introduction

 Dentin hypersensitivity is a common painful condition among adults, with approximately one in six patients suffering from it. It is also a complex phenomenon that causes psychological and physiological changes.^1,2^ Pain in dentin sensitivity is acute and fast and varies in intensity. It is mostly triggered by thermal (cold and/or heat), mechanical or tactile, chemical (acid and sweet), and bacterial stimuli.^3^ Numerous theories have been proposed to explain dentin sensitivity; the most widely accepted theory is dentin hydrolytic conduction, also known as the hydrodynamic theory.^4^

 There are various therapeutic options for treating dentin sensitivity in the literature. This variation has led to confusion among dentists, making them unable to provide effective and definitive treatment.^3,5^

 The hydrodynamic theory has suggested methods for blocking dentinal tubules using various fluoride products, such as fluoride-containing toothpastes and mouthwashes, specialized use of fluoride varnishes, dentin adhesives, corticosteroids, and silver nitrate.^6-8^ Dentinal tubules can be occluded at the orifice or inside the tubules. It is assumed that closing the gap between the tubules or sealing the tubules is the most reliable way for long-term success. Sediments or barriers formed by desensitizing substances might be removed between successive applications or shortly after use due to abrasive wear by a toothbrush or dissolved due to thermal cycles in the oral environment, resulting in short-term desensitizing effects.^6^

 Sodium fluoride is one of the most commonly used desensitizing agents to treat dentin sensitivity, which is used in a variety of ways. Sodium fluoride application is suggested to increase the time of fluoride function in contact with dentinal tubules, decreasing the sensitivity.^7^

 Bioactive casein phosphopeptide-amorphous calcium phosphate (CPP-ACP) is a form of calcium phosphate stabilized by milk casein protein phospho-peptides.^9^ The compound is also commercially available to treat dentin sensitivity.^10^

 Many previous studies have only examined the effect of selective desensitizing materials on dentin, providing evidence for deposition durability within dentinal tubules and thus their potential for decreasing sensitivity over time, while the greatest challenge in treating dentin sensitivity is to gain long-term impact.^11-13^ This study aimed to compare the penetration and durability of two dentin desensitizers, calcium fluoride varnish and CPP-ACP, using electron microscopy.

## Methods

###  Preparation of specimens 

 The in vitro study compared two desensitizing agents for three periods of thermal and abrasive conditions. The specimens were prepared from 10 freshly extracted healthy adult human premolar teeth (20-40 years old without decay) extracted for orthodontic or surgical reasons, stored in standard laboratory conditions. All the tooth samples were immersed in deionized water and ultrasonically cleaned of gross debris. All the specimens were examined for any defects, such as cracks and holes, under a light microscope and stored for a maximum of one month until they were used.

 The surface enamel was removed by grinding with a 600-grit disk for 20 seconds. The teeth were sectioned in the same direction using a water-cooled diamond saw. Each tooth was mounted on a resin block, one-third of the occlusal crown was removed by a cutting machine, and two incised dentinal discs measured 1 mm above the CEJ. Two dentin discs were prepared from each tooth,^11^ and each disk was cut into three sections.

 The 60 samples were individually placed in an ultrasonic apparatus for 30 seconds and then washed with normal saline solution. The samples were etched on the surface with 17% EDTA etchant with a microbrush for two minutes and then rinsed with distilled water for 30 seconds to exposes the dentinal tubules’ orifices, resembling the exposed dentin pattern (simulating dentin hypersensitive cervical regions).^14^

 Desensitizing agents were applied in two groups as follows:

- Group 1: Sodium fluoride varnish and calcium fluoride single-dose Bi-fluoride 10 (Voco, Germany) was applied. According to the manufacturer’s instructions, the surface of the sample was air-dried. A thin layer of varnish was applied to the surface. After 10‒20 seconds, it was dried with air.
- Group 2: A varnish containing fluoride and CPP-ACP (MI Paste) (GC, Melbourne, Australia) was applied to the dentin surfaces.

The two groups were examined by scanning electron microscopy (SEM) for sample surface quality and penetration depth.

###  Thermal cycling and toothbrush abrasion

 Since the penetration depth of the agent could not be the only criterion for the actual durability, the following steps were taken to evaluate the durability of agents:

1. One-third of the samples were placed in a thermocycling device (Delta Tpo2, Iran) to simulate the ambient temperature conditions. Both groups were immersed in two baths of distilled water (5 ºC and 55 ºC) for 15 seconds. The specimens were immersed in 23°C water for 5 seconds. A total of 150 cycles were repeated for 15 days (10 cycles/day).
2. The samples were also placed in a toothbrush simulator (Syesh, Iran) to simulate the normal brushing force. They were immersed in a toothpaste solution (Crest 7 Complete, Germany) diluted 1: 1 with an average force of 150 g, applied with a toothbrush of moderate stiffness. A new toothbrush and toothpaste solution were used for each group.^15^

For one-third of the samples, 300 thermal cycles equivalent to 30 days and 600 cycles of toothbrush simulation were completed.

###  SEM analysis

 The specimens were mounted on SEM stubs and sputter-coated with an approximately 200-A˚ gold layer and examined under SEM (Philips, ESEM XL30) at magnifications of × 1200 to × 2000 for tubular occlusion. The specimens from each group were axially fractured and re-sputter-coated with gold and studied to indicate the agent’s depth of penetration. The surface coverage percentage and the extent of tubular occlusion were measured by ImageJ software (1.52a USA 2017).

**Figure 1:**
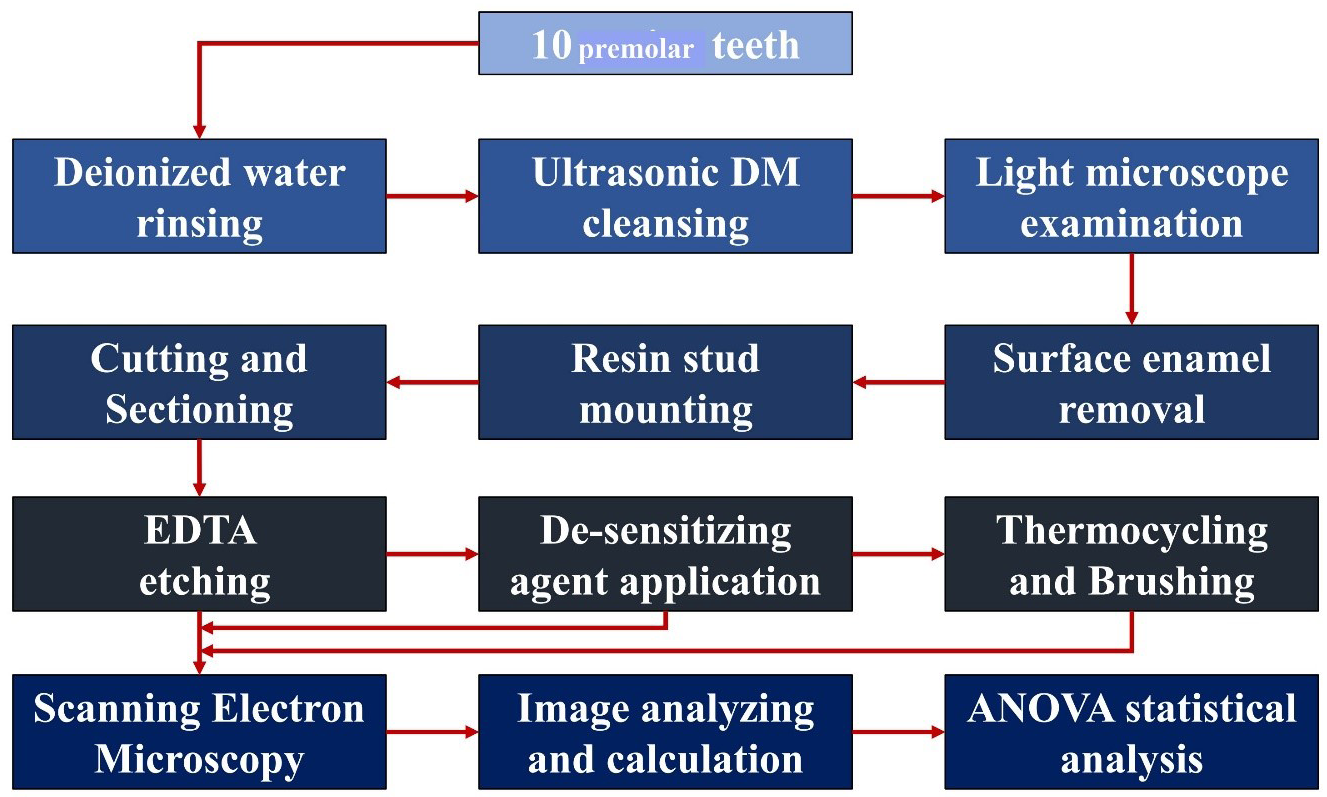
A schematic representation of experimental procedures

 The samples were classified into four groups according to the measured percentage for surface coverage:

- A: The dentinal tubules were somewhat occluded, and the orifices could be seen (surface coverage: ≤ 60%)
- B: The dentinal tubules were mostly occluded, with no signs of agent deposits on the surface (surface coverage: 60‒90%).
- C: The dentinal tubules were mostly occluded, and the surface of the dentin specimen was relatively covered with the agent (surface coverage: 90‒98%).
- D: All the dentinal tubules were completely occluded, and the surface was completely covered with the agent (surface coverage: > 98%). In this case, crystalline-like deposits completely covered the dentin surface. In some areas, there were clusters of agents (high-quantities which could be seen in white). Figure 1 presents the four classifications.

 After imaging the cross-sectional view of the tubules, the specimens were fractured in liquid nitrogen, and micrographs of longitudinal tubular sections were captured. The dispositioned material in dentin tubules was categorized using the following expressions^16^:

- Plug: Initial infiltration of the agent into the entrance of tubules.
- Globular structure: The deposition of the agent within the tubule beyond the primary penetration.
- Envelope formation: It is seen in cases where the infiltrated agent is not dense and is disconnected.

###  Measurements and statistical analysis

**Figure 2:**
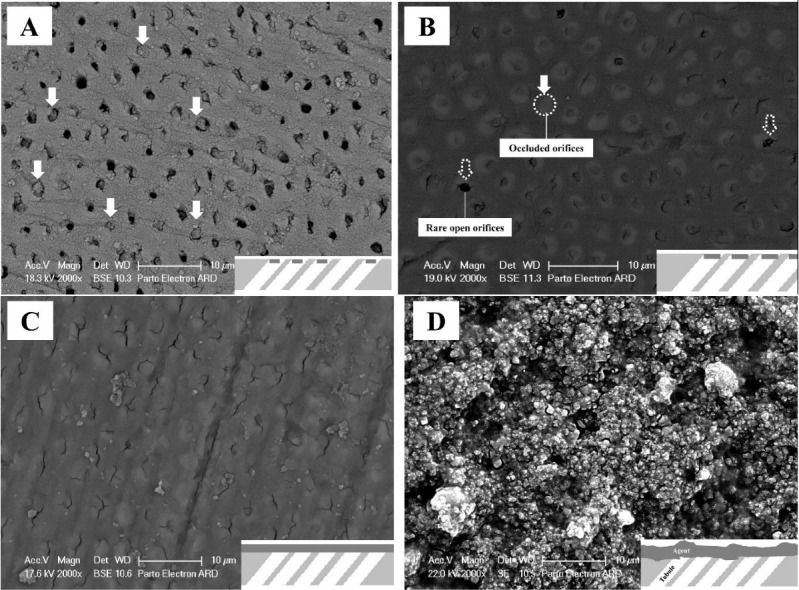
Surface classification of samples (A, B, C, and D) based on the dentin surface coverage percentage of desensitizing agents. Details and schematic representation of the occlusion are shown as inlets

 The maximum agent penetration depth, the thickness of the formed plug, and the surface distance of the formed plug were measured by ImageJ software (1.52a USA 2017) in 20 tubules of each specimen,^17^ and the mean was calculated as the data. The data were analyzed using two-way ANOVA and Mann-Whitney test. SPSS 20 was used for statistical analyses. The acceptable error in the study was considered at 0.05. A schematic process chart is shown in Figure 2.

## Results

 The results of SEM images are reported in two parts: cross-section and longitudinal. Different parameters are examined in each part.

###  Cross-section

**Figure 3:**
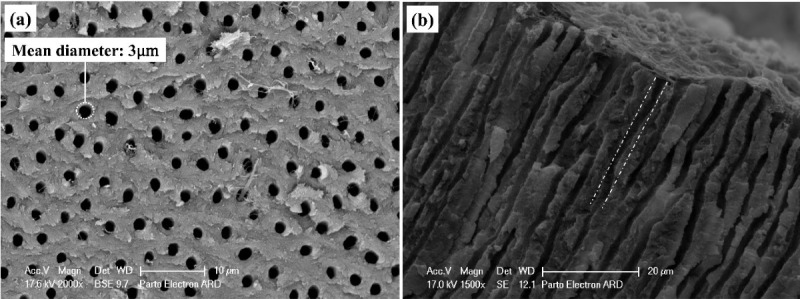
Electron microscope image ( × 1200) of dentin surface etched with 17% EDTA for two minutes to remove the smear layer and simulate open orifice of sensitive dentinal tubules at (a) surface and (b) cross-section

 The initial preparation of samples created open tubular orifices without residual mass, deposits, and debris. Figure 3 presents the SEM micrograph of a cleansed and etched sample.

**Figure 4:**
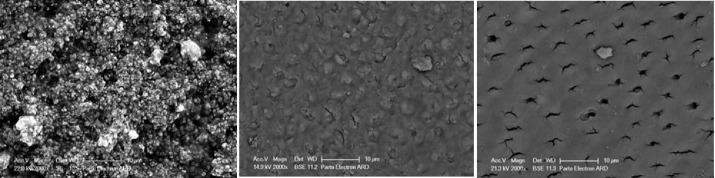
Surface micrograph of MI Paste samples. Left to right: Immediately after application, after 15 days, and after 30 days

**Figure 5:**
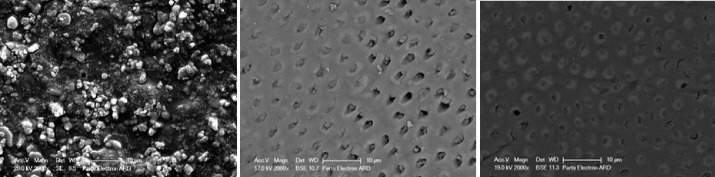
Surface micrograph of Bi-fluoride varnish samples. Left to right: Immediately after application, after 15 days, and after 30 days

 Two-way ANOVA showed that the effect of the agent type on the surface coverage percentage for each agent was not significant (*P* = 0.23); however, the effect of time (*P* < 0.001) and the interaction of group and time (*P* = 0.03) were significant (Table 1). Immediately after application, the samples exhibited a “D” surface coverage pattern, which changed by thermocycling and toothbrush abrasion (Figures 4 and 5).

**Table 1:** Mean percentage of surface coverage by agent in the two groups at different times

**Sample**	**CPP-ACP**		**Bi-fluoride varnish**	
	**Mean**	**Standard deviation**	**Mean**	**Standard deviation**
Immediately after application of the agent	99.6	0.5	100	0
15 days after thermal cycles (300 cycles) and toothbrush abrasion	87.9	5.5	85.2	13.1
30 days after thermal cycles (600 cycles) and toothbrush wear	84.9	5.2	93.4	5.1

**Figure 6:**
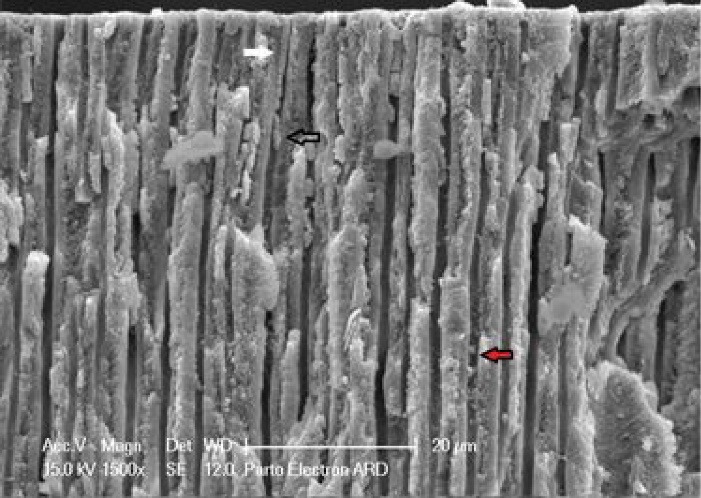
Agent deposition along dentinal tubules. Plug (white arrow), globular structure (red arrow), and envelope formation (black arrow)

Tables 2 and 3 present the distribution of surface coverage patterns in the two groups and surface coating patterns in the two groups. Mann-Whitney test showed no significant difference between the two groups in surface coating patterns (*P* = 0.47) (Figure 6).

**Table 2:** Distribution of surface coverage patterns in the two groups

	**Surface coverage patterns**	**A**	**B**	**C**	**D**
MI Paste	Immediately after application of the agent	0	0	0	10
	15 Days after thermal cycles (300 cycles) and toothbrush abrasion	3	4	3	0
	30 Days after thermal cycles (600 cycles) and toothbrush wear	2	5	3	0
Bi-fluoride varnish	Immediately after application of the agent	0	0	0	10
	15 Days after thermal cycles (300 cycles) and toothbrush abrasion	2	5	2	1
	30 Days after thermal cycles (600 cycles) and toothbrush wear	0	5	3	2

**Table 3:** Distribution of surface coating pattern in two groups

**Pattern**	**CPP-ACP**		**Bi-fluoride varnish**		***P*** ** value**
	**Counts**	**Percent**	**Counts**	**Percent**	
A	4	13.3	2	6.7	0.47
B	9	30	10	33.3	
C	7	23.4	5	16.7	
D	10	33.3	13	43.4	

 Tubular occlusion values are presented in Table 4. Two-way ANOVA showed that the effect of group (*P* < 0.001) and time (*P* < 0.001) on tubule obstruction was significant; however, the interaction between group and time was not significant (*P* = 0.09). In other words, the mean of tubular obstruction was significantly different between the three time intervals, and this difference was almost identical in the two groups.

**Table 4:** Mean tubular occlusion in the two groups at different time periods

**Samples**	**CPP-ACP**		**Bi-fluoride varnish**	
	**Mean**	**Standard deviation**	**Mean**	**Standard deviation**
Immediately after application of the agent	98.2	1.1	100	0
15 Days after thermal cycles (300 cycles) and toothbrush abrasion	69.5	12.5	79.9	15.9
30 Days after thermal cycles (600 cycles) and toothbrush wear	77.6	7.9	92.2	5.8

###  Longitudinal

**Figure 7:**
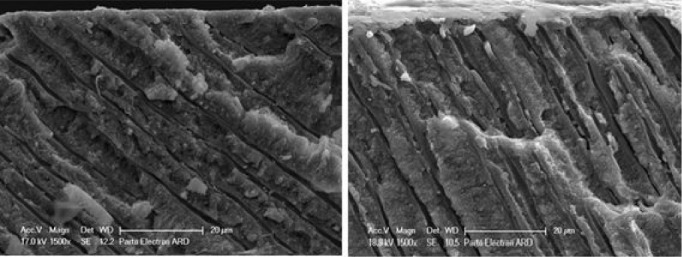
Longitudinal sections of dentin specimens. Right: Bi-fluoride varnish agent, left: MI Paste

 The longitudinal section of the tubules showed the depth of agent penetration into the orifices. Table 5 presents the mean depth of agent penetration in the two groups at different time intervals. Two-way ANOVA showed that the effect of group (*P* < 0.001), time (P < 0.001), and group-time interaction (*P* = 0.005) were significant on the depth of agent penetration. In other words, the mean depth of material penetration was significantly different between the three time intervals, and this difference was not the same in the two groups (Figure 7).

**Table 5:** Mean depth of agent penetration in the two groups at different time periods

**Samples**	**CPP-ACP**		**Bi-fluoride varnish**	
	**Mean**	**Standard deviation**	**Mean**	**Standard deviation**
Immediately after application of the agent	30.9	2.3	30	2.2
15 Days after thermal cycles (300 cycles) and toothbrush abrasion	46.6	3.1	46.6	3.1
30 Days after thermal cycles (600 cycles) and toothbrush wear	32.8	11.2	44.3	13.2

Table 6 presents the patterns of agent penetration into the dentinal tubules. The probability of plug formation was studied at tubule orifices and then controlled along the tubule (middle). The globular structure of occlusion was categorized into circular and elliptical shapes based on morphology. The presence of different occlusion types is reported as seen ( + ) and not seen (-).

**Table 6:** Distribution of surface coverage patterns in the two groups

		**Plug**		**Globular**	
**surface coverage patterns**		**Orifice**	**Middle**	**Circular**	**Elliptic**
MI Paste	Immediately after application of the agent	+	+	-	NA
	15 days after thermal cycles (300 cycles) and toothbrush abrasion	+	+	+	NA
	30 days after thermal cycles (600 cycles) and toothbrush wear	+	-	-	+
Bi-fluoride varnish	Immediately after application of the agent	+	+	+	+
	15 days after thermal cycles (300 cycles) and toothbrush abrasion	+	+	+	+
	30 days after thermal cycles (600 cycles) and toothbrush wear	+	+	+	NA

NA: Not seen.

 Plug distance from the surface is another factor that can indicate the capability of the agent for tubular occlusion. Table 7 shows the changes in this distance for agents at different time intervals. Two-way ANOVA showed that the effect of group (*P* < 0.001), time (*P* < 0.001), and the interaction between agent type and time (*P* = 0.002) on the distance between the plugs formed was significant. In other words, the mean distance between plugs formed from the surface was significantly different between the three time intervals, and this difference was not the same in the two groups. In addition, the increase in surface area of the plug in the varnish group was more than the CPP-ACP group.

 Plug thickness also shows the quality of agents and time intervals in occluding the tubules. Table 7 also shows plug thicknesses in the two groups at different time intervals. Two-way ANOVA showed that the effect of group, time, and the interaction of agent type and time on the plug thickness was significant (*P* < 0.001). In other words, the average thickness of the plugs formed was significantly different between the three time intervals, and this difference was not the same in the two groups.

**Table 7:** Plug distance and thickness from surface in the two groups at different time periods

	**CPP-ACP**		**Bi-fluoride varnish**	
	**Mean**	**Standard deviation**	**Mean**	**Standard deviation**
**Plug distance**				
Immediately after application of the agent	15.9	2.9	17	4.03
15 Days after thermal cycles (300 cycles) and toothbrush abrasion	22.9	3.2	38.3	7.8
30 Days after thermal cycles (600 cycles) and toothbrush wear	27.7	11.2	39.5	4.1
**Plug thickness**				
Immediately after application of the agent	14.9	0.9	13	0.8
15 Days after thermal cycles (300 cycles) and toothbrush abrasion	14.02	3.3	8.2	1.4
30 Days after thermal cycles (600 cycles) and toothbrush wear	5.1	0.7	4.9	0.9

## Discussion

 Despite the variety of available therapies, increased dentin sensitivity is still a chronic dental problem with an unfavorable prognosis. It is possible to transfer the stimulation to the nerve terminals of odontoblasts by reducing fluid movement within the dentinal tubules by narrowing or blocking the tubular opening.^17-19^ To simulate sensitive dentin surfaces, all the specimens were etched with 17% EDTA before applying the anesthetic agent. This step was performed to ensure the removal of the smear layer or smear plug that might be mistaken in the images for the therapeutic agent used. Also, many wide-open tubules have been shown in sensitive dentin. Therefore, the etching process helps make the specimens similar to the sensitive dentin.^12^

 The mean percentage of surface coverage by agents was significantly different between the three time intervals, and this difference was not the same in the two groups (Table 7). The percentage of surface coverage by the agent significantly reduced 15 days after applying the thermal cycles and toothbrush abrasion compared to immediately after the application. It increased again 30 days after the application of the thermal cycles and toothbrush abrasion. Both types of agents, immediately after application to the dentin, exhibited a high percentage of surface coating; therefore, the pattern of surface coatings immediately after application was like a “D” pattern. This means that both agents were capable of occluding the dentinal tubules, although this coverage was higher with the Bi-fluoride agent. Bi-fluoride varnish dries quickly and adheres to the surface compared to the paste-like nature of MI Paste. However, over time, due to the abrasion of the toothbrush, the surface pattern changed. After 15 days of applying 300 cycles of heat and toothbrush simulator on the surface of the specimens, the created heat reduced the surface coverage seen in the micrographs for both agents. After 30 days, the samples from the Bi-fluoride group exhibited an increase in the percentage of surface coverage. However, in the MI Paste group, the surface coverage continued to decrease. It can be stated that fluoride ions released from the Bi-fluoride 10 varnish on the surface of the tooth transformed into calcium fluoride. Calcium fluoride is insoluble and has a higher molecular weight than sodium fluoride (5.45 A˚ vs. 4.62 A˚).^13^

 The trend observed in this study is similar to that reported by Canali et al.^20^ In their study, they evaluated the degree of tubular occlusion after using different agents (two types of varnish and one type of paste and one desensitizing gel) and the application of erosion and abrasion challenges, at three intervals of one, four, and seven days. The results showed that although all the agents could reduce dentin permeability immediately after application, only varnishes can maintain the tubular blocking properties after acidic materials’ attack and toothbrush wear.

 Examination of the micrographs obtained in this study showed that in both agents, the tubular occlusion decreased with time and heat and brushing abrasion, which might be attributed to the increased fluidity of the paste agent, such as MI Paste, and its further penetration into the tubules. The Bi-fluoride agent keeps its adherence capabilities by continuing conversion to calcium fluoride, leading to further tubular occlusion.^21^

 The permeability relationship between surface and deposition inside the tubules and dentin sensitivity is not simple. The amount of deposition within the tubule and the quality of these depositions, including their density, porosity, and depth of penetration, are significant. In addition, the penetration depth of an agent cannot guarantee its survival and durability. At a high penetration depth, it might rapidly lose its efficiency in a short time or as a result of other affecting factors. Al-Saud et al^12^ investigated the ability of Nd:YAG lasers and four types of dentin agents (Gluma, Tenure Quick, VivaSens, and Quelle desensitizer) to block dentinal tubules in vitro in conditions simulated like oral conditions. They reported that both lasers and desensitizing agents are capable of occluding the tubules; however, the deposition of the bonding agent on the dentin surface is not very firm, so that the durability of the desensitizing agents in the dynamic oral environment can be questionable.

 Therefore, in the present study, in addition to investigating the depth of initial agent penetration into dentinal tubules, the depth of penetration after thermal cycles and toothbrush abrasion were also studied.

 The examination of tubules’ longitudinal sections showed that the mean penetration depths for Bi-fluoride and MI Paste were 30 μm and 30.9 μm, respectively. However, depth changes over time were significantly greater in the varnish group than in the CPP-ACP group, indicating that in the first 15 days, the tubules diameter increases due to heating (expansion), increasing the penetration depth of the material. Then in the second 15 days, the penetration depth decreases due to a decrease in tubular capillary pressure and outward movement of this agent.

 Limited laboratory studies have been conducted to measure the penetration depth of desensitizing agents into dentinal tubules. Tosun et al^13^ evaluated the penetration depth of Clinpro Varnish (5% sodium fluoride + tricalcium phosphate) in dentin samples after only five days of pH cycling by electron microscopy. The reported mean penetration depth was different from our results that can be attributed to differences in dentin specimens prepared from molar teeth. Tosun’s study did not examine the penetration depth of agents, either using a toothbrush or thermocycling.

 Our results of the penetration pattern of both desensitizing agents into dentinal tubules (Table 6) showed that Bi-fluoride constituted a large number of plugs at the beginning of application that could also be seen along the tubule. Many spherical and elliptical globular patterns were detected. Thermal and abrasive treatments showed that the size of the plugs and globular structures decreased. In the case of MI Paste, first, there was good infiltration of the agent with the plug formation, but the number of globular structures was lower than Bi-fluoride. Therefore, it can be concluded that the cause of most of the globules in the varnish group is the dryer nature of the material.

 After 15 days of paste agent application, the thickness of the plugs formed in the tubules decreased by less than half, which could be due to material degradation. The results showed that the mean surface distance of the plug in the CPP-ACP group increased over time. It seems that the removal of the applied agent increased the plug distance formed at the sample surface. In the varnish group, 15- and 30-day plugs were larger than those immediately after application, and there was no significant difference between 15 and 30 days. To explain, in the Bi-fluoride material because of the adhesion of the agent, there was no further removal of the agent between 15 days and 30 days. The mean thickness of the plug formed in the varnish group decreased over time. In the CPP-ACP group, the mean thickness of the plug 30 days after thermal cycles and toothbrush abrasion was less than 15 days and immediately after application. There was no significant difference between 15 days and immediately after application. Once the agent is used, the lifetime of the MI Paste material is between one and two weeks. When the material penetration into the tubule suddenly decreases, the agent is damaged, and it does not have sufficient opportunity to form hydroxyapatite in significant amounts to occlude dentinal tubules. On the other hand, good adhesion of Bi-fluoride to the surface and inside the tubules results in a linear decrease in the uniform thickness of the plug formed within the tubule after abrasion and thermal cycles.^22^

 No laboratory study has evaluated the durability of desensitizing agents under simulated mouth conditions; therefore, the results of clinical studies are reviewed instead. Jain et al^23^ reported a 58.86% decrease in dentin sensitivity due to thermal and tactile stimuli immediately after varnish application. Dentin sensitivity decreased by half after 15 to 60 days due to toothbrush abrasion. In another clinical study, Corona et al^24^ reported that the effect of sodium fluoride on pain relief in patients with dentin sensitivity decreased from day 15 to day 30. However, the efficacy of the sodium fluoride varnish and the low-power laser to reduce dentin sensitivity was similar. Kara and Orbak^25^ compared the effect of Nd-YAG laser and fluoride varnish on decreasing dentin sensitivity. Patients treated with fluoride varnish exhibited significant pain reduction after two weeks. The fluoride varnish was significantly more effective for treating dentin sensitivity than laser treatment. Hedge et al^26^ compared the performance of Bi-fluoride varnish and APF with iontophoresis, reporting that in cases of Bi-fluoride varnish, 40% of areas of teeth exhibited complete remission of symptoms up to eight weeks, simultaneous with the initiation of desensitizing therapy. The relative decrease in treatment performance was observed at 4- and 8-week follow-ups.

 A comparison of the results of the above studies and the present study showed that fluoride varnish could immediately decrease pain, which is effective for up to two weeks.

## Conclusion

 MI Paste exhibited less durability than Bi-fluoride in oral conditions and degraded after 15 days. It is also possible to expect more effective and reliable results from a varnish that is more adherent to the dentin surface if used only once. Bi-fluoride exhibited a more immediate effect on tubular obstruction than MI Paste.

## Acknowledgments

 Authors would like to thank Dr. Navid Hosseinabadi from Islamic Azad University/Shiraz branch (Department of Materials Engineering and Metallurgy) for his assistance in analyzing SEM micrographs.

## Competing Interests

 The authors declare no competing interests with regards to authorship and/or publication of this article.

## Ethical Approval

 This research was approved by the Research Ethics Committee of Islamic Azad University of Isfahan under the code IR.IAU.KHUISF.REC.1398.124.

## Funding

 Nil.
